# Inhibition of MIR4435-2HG on Invasion, Migration, and EMT of Gastric Carcinoma Cells by Mediating MiR-138-5p/Sox4 Axis

**DOI:** 10.3389/fonc.2021.661288

**Published:** 2021-08-31

**Authors:** Li-Fei Gao, Wei Li, Ya-Gang Liu, Cui Zhang, Wei-Na Gao, Liang Wang

**Affiliations:** ^1^The Third Department of General Surgery, Cangzhou Central Hospital, Cangzhou, China; ^2^The Second Department of General Surgery, Cangzhou Central Hospital, Cangzhou, China; ^3^The Fourth Department of Endocrinology, Cangzhou Central Hospital, Cangzhou, China

**Keywords:** MIR4435-2HG, miR-138-5p, Sox4, gastric carcinoma, EMT, ceRNA

## Abstract

**Background:**

The previous investigations have identified that long non-coding RNA (lncRNAs) act as crucial regulators in gastric carcinoma. However, the function of lncRNA MIR4435-2HG in the modulation of gastric carcinoma remains elusive. Here, we aimed to explore the role of MIR4435-2HG in gastric carcinoma.

**Method:**

The Cancer Genome Atlas (TCGA) and Gene Expression Omnibus (GEO) were applied to select the differently expressed lncRNAs in gastric carcinoma. The qRT-PCR was applied to analyze MIR4435-2HG expression in carcinoma tissues and cell lines. The effect of MIR4435-2HG on proliferation, invasion, migration, and apoptosis of gastric carcinoma cells was detected by Cell Counting Kit-8 (CCK-8) assays, transwell assays, and flow cytometry *in vitro*. A subcutaneous tumor model was constructed to examine the tumor growth of gastric carcinoma cells after knocking out MIR4435-2HG. RNA immunoprecipitation and luciferase reporting assays were applied to evaluate the interaction of MIR4435-2HG, miR-138-5p, and Sox4.

**Results:**

The bioinformatics analysis based on TCGA and GEO databases indicated that MIR4435-2HG was obviously elevated in gastric carcinoma samples. The qRT-PCR analysis revealed that MIR4435-2HG was upregulated in clinical gastric carcinoma tissues and cells. The high expression of MIR4435-2HG is associated with the poor survival rate of patients. The knockout of MIR4435-2HG could repress the proliferation, invasion, migration, and epithelial–mesenchymal transition (EMT) and accelerate the apoptosis of gastric carcinoma cells. Moreover, the deletion of MIR4435-2HG was able to attenuate the tumor growth *in vivo*. Mechanically, we identified that MIR4435-2HG enhanced Sox4 expression by directly interacting with miR-138-5p as a competitive endogenous RNA (ceRNA) in gastric carcinoma cells, in which Sox4 was targeted by miR-138-5p.

**Conclusion:**

MIR4435-2HG is elevated in gastric carcinoma cells and contributes to the growth, metastasis, and EMT of gastric carcinoma cells by targeting miR-138-5p/Sox4 axis. MIR4435-2HG may be applied as a potential therapeutic target in gastric carcinoma.

## Introduction

Gastric carcinoma is a common malignancy of the digestive tract system. It has been indicated that the progression of gastric carcinoma is triggered by the interaction of various environmental carcinogenic factors (1, 2). A recent epidemiological survey suggests that more than 980,000 gastric carcinoma cases are confirmed as gastric carcinoma every year, and more than half of them are Asian (3). However, many patients have reached the middle and late stages with malignant hyperplasia after being confirmed, accompanied by extensive infiltration and distant metastasis (4, 5). Epithelial–mesenchymal transition (EMT) is closely correlated with the metastasis in gastric carcinoma cells (6, 7). The previous studies have demonstrated that the progression of EMT was induced in invasion and metastasis of gastric carcinoma cells (8), but the mechanism of EMT is still under exploration.

Long non-coding RNAs (lncRNAs) are a type of non-coding RNA transcripts longer than 200 nucleotides (nt) in length, with limited protein-coding capacity, and have shown critical functions in various cellular processes (9–12). Moreover, lncRNAs participate in the tumorigenesis and development of cancers by affecting different biological pathways, and their regulatory roles and clinical significance are gradually confirmed by more and more investigations (13–15). For instance (16), lncRNA PTAR preserves ZEB1 overexpression by keeping miR-101 spongy and accelerating EMT, invasion, and metastasis of ovarian carcinoma. Another survey has reported that (17) lncRNA RNA MEG-3 inhibits the progression, invasion, and migration of gastric carcinoma through EMT regulation.

MIR4435-2HG is a poorly studied lncRNA and has been found to modulate cancer progression in ovarian carcinoma (18), glioma (19), and lung carcinoma (20). However, its role in gastric carcinoma is still unreported. It has been reported that MIR4435-2HG is involved in the modulation of gastric cancer by the activation of Wnt/β-catenin signaling (21), but the clinical significance and the effect of MIR4435-2HG on EMT in gastric carcinoma remain unclear. In this study, the differently expressed lncRNAs in gastric carcinoma were analyzed in Gene Expression Omnibus (GEO) and The Cancer Genome Atlas (TCGA), and we identified that MIR4435-2HG was elevated in gastric carcinoma, indicating that MIR4435-2HG might play a crucial function in gastric carcinoma cells.

Therefore, this research aimed to explore the role of MIR4435-2HG in gastric carcinoma, especially its function in EMT. This investigation may contribute to lncRNA-oriented diagnosis and treatment of gastric carcinoma.

## Materials and Methods

### Materials

From January 2012 to January 2014, 60 patients with gastric carcinoma were obtained. During the intervention, the tumor tissues and adjacent tissues were collected, sent to laboratory for detection, and placed at −80°C. All patients were confirmed as gastric carcinoma by pathological examination. The patients had not received anti-tumor treatment before intervention, and the estimated survival was more than 1 month. This study conformed to the Medical Ethics Committee of Cangzhou Central Hospital and the Declaration of Helsinki. The patients provided their written informed consent to participate in this study. All patients could cooperate with the research (22).

### TCGA and GEO Analysis

#### TCGA

We logged into TCGA (https://portal.gdc.carcinoma.gov), downloaded the transcript data of gastric adenocarcinoma, organized the files into matrix files, and extracted MIR4435-2HG data to test their differences. A total of 373 samples were found, including 30 normal controls and 343 tumors.

#### GEO

We logged into https://www.ncbi.nlm.nih.gov/gds, searched gastric carcinoma and lncRNA-correlated chips, selected GSE109476 chip after primary screening, downloaded annotated files, merged them into matrix files, and extracted MIR4435-2HG data from them to test the differences. Twenty samples were found, including 10 normal controls and 10 tumors.

### Cell Cultivation

HGC-27, SGC-7901, MGC-803 and AGS, and GES-1 were collected from the CBTCC and cultivated in Roswell Park Memorial Institute (RPMI)-1640, which contains 10% fetal bovine serum (FBS), at 37°C with 5% CO_2_.

### Cell Transfection

When the growth of gastric carcinoma cells reached 50%–70%, 50 nM of si-RNA (si-MIR4435-2HG#1, 2, 3) negative control (si-NC) was transfected into gastric carcinoma cells. And 20 nM of miR-138-5p mimics or miR-NC mimics, anti-mir-138-5p, or anti-miR-NC; and 10 ng of Sox4 (SRY-box transcription factor 4) overexpression plasmid (pcDNA-Sox4) or negative plasmid (vector-NC) were applied for transfection using Lipofectamine 2000. After 48 h, cells were harvested for qPCR analysis. A recombinant lentiviral vector (SH-MIR4435-2HG) was also constructed for tumorigenesis in nude mice. SGC-7901 cells were infected with lentivirus-sh-MIR4435-2HG or interfered with different multiplicities of infection in a medium containing polybrominated substances (8 μg/ml). After 24 h, puromycin was applied to selectively transfect positive cells at a final concentration of 1 μg/ml.

### qRT-PCR detection

Total RNA was obtained from the collected samples. Then, reverse transcription was conducted, and cDNA was obtained for subsequent tests. PCR amplification was applied using SYBR Green. The quantification of lncRNA and miRNA (miR) was conducted using 2^−ΔΔCt^. GAPDH and U6 were applied as internal references for lncRNA/mRNA and miRNA (23). The sequence of primers is shown in **Table 1**.

**Table 1:** Primer sequence.

Gene	Upstream primer	Downstream primer
*MIR4435-2HG*	5′-TGATAAAGGGCTCTGAAAGC-3′	5′-CACGATGCCTTCACCAGTGT-3′
*MiR-138-5p*	5′-AGCTGGTGTTGTGAATCAGGCCG-3′	5′-TGGTGTCGTGGAGTCG-3′
*Sox4*	5′-GTGAGCGAGATGATCTCGGG-3′	5′-CAGGTTGGAGATGCTGGACTC-3′
*E-cadherin*	5′-GGCGCCACCTCGAGAGA-3′	5′-TGTCGACCGGTGCAATCTT3′
*Vimentin*	5′-GGCTCAGATTCAGGAACAGC-3′	5′-CTGAATCTCATCCTGCAGGC-3′
*Fibronectin*	*5′-AGACCATACCTGCCGAATGTAG-3′*	*5′-GAGAGCTTCCTGTCCTGTAGAG-3′*
*GAPDH*	5′-CTGACTTCAACAGCGACAC-3′	5′-TAGCCAAATTCGTTGTCATAC-3′
*U6*	5′-CTCGCTTCGGCAGCACA-3′	5′-AACGCTTCACGAATTTGCGT-3′

### Western Blotting

Transfected cells were obtained to extract the total cell protein using radioimmunoprecipitation assay (RIPA) buffer (Thermo, USA), and the concentration was tested. A 30-µg protein was separated using 12% sodium dodecyl sulfate–polyacrylamide gel electrophoresis (SDS-PAGE). The protein was moved to membrane; sealed in 5% skim milk at 37°C for 1 h; incubated at 4°C; added with main antibodies, such as E-cadherin, vimentin, fibronectin, Sox4, and β-actin; incubated for 12 h; and then added with horseradish peroxidase (HRP)-bound goat anti-rabbit IgG monoclonal secondary antibody or rabbit anti-mouse IgG monoclonal secondary antibody at 37°C for 2 h. Proteins were visualized using SuperSignal West Pico.

### Cell Proliferation

MTT method was applied to test the proliferation, and the specific steps were as follows: the transfected cells were obtained, resuspended to 5 × 10^3^ cells/well, and inoculated into 96-well plates at 37°C. MTT (5 mg/ml, 20 µl) was put into each well at 0, 24, 48, and 72 h, and the plates were cultivated for 4 h. Thereafter, the culture medium was removed, and 100 μl of dimethyl sulfoxide was added. The proliferation was tested at 560 nm using a microplate reader.

### Cell Invasion and Migration

Cells were obtained in Matrigel-coated and uncoated transwell inserts, and the invasion and migration were tested. The specific steps were as follows: the cells were changed to 1 × 10^4^ and transferred to the upper chamber including serum-free Dulbecco’s modified Eagle’s medium (DMEM) and the lower chamber including 5% FBS. After 24 h, the cells in the upper chamber were wiped with cotton swabs; and the lower chamber was fixed in 4% paraformaldehyde for 15 min, dyed with 0.1% crystal violet dye (Sigma-Aldrich), and placed at 37°C for 20 min. Then, the cells were counted on three randomly selected views using an optical microscope (Olympus Corporation).

### Apoptosis

Cell apoptosis was tested using flow cytometry, and the specific scheme was as follows: after 48-h transfection, cells were obtained, rinsed with phosphate-buffered saline (PBS), and fixed with 70% ethanol. The fixed cells were rehydrated in PBS for 10 min, incubated in RNase A (1 mg/ml) at 37°C for 30 min, dyed with PI/RNase, and finally tested using flow cytometry (FACScan). All samples were tested in triplicate.

### Double Luciferase Report

MIR4435-2HG or SOX4 mRNA fragment was synthesized and inserted into psiCHECK-2 luciferase reporter vector. MiR-138-5p mimetic or NC with reporter gene plasmid was co-transfected into HEK293T cells. After 48 h, luciferase activity was tested using Dual-luciferase Reporter.

### RNA Immunoprecipitation

RNA immunoprecipitation (RIP) was tested using Pierce™ Magnetic RNA-Protein Pull-Down. The cell lysate was cultivated with RIP buffer, which was conjugated with anti-Ago2 antibody or normal mouse IgG. Immunoprecipitated RNA was purified and tested using qRT-PCR.

### Tumor Formation in Nude Mice

Naked mice were obtained from Charles River. Ten male athymic nude mice, aged 5–6 weeks, were fed by the Animal Experimental Center. The cell density was changed to 1 × 10^7^ cells/ml. A total of 20 µl of suspension was subcutaneously inoculated into the armpit of nude mice. After 28 days, nude mice were euthanized (asphyxiated with CO_2_), and then the tumor mass was tested. At the 7th, 14th, 21st, and 28th days of modeling, the tumor volume was tested, and the growth curve was visualized. The tumor volume was tested as follows: (a × b^2^)/2. This experiment conformed to the Animal Medical Ethics Committee of Cangzhou Central Hospital and met institutional animal care and use committee (IACUC) standards of Cangzhou Central Hospital.

### Statistical Analysis

SPSS20.0 was applied for statistical analysis and GraphPad 7 for visualizing the required pictures. Measurement data were represented as (mean ± SD) and compared by independent-samples *t*-test. Counting data were represented by (rate) and compared by the chi-square test. One-way ANOVA was applied for comparison among groups and least significant difference (LSD) *t*-test afterward. Multiple time points were tested using repeated measurement variance, which was expressed by F. Back testing applied Bonferroni. Pearson’s test was applied to analyze the correlation of gene expression; the Kaplan–Meier (K-M) survival curve was used to visualize the 5-year survival, which was tested using log-rank test. When p < 0.05, there was statistical difference.

## Results

### MIR4435-2HG Elevated in Gastric Carcinoma With Poor Prognosis

**Figure 1:**
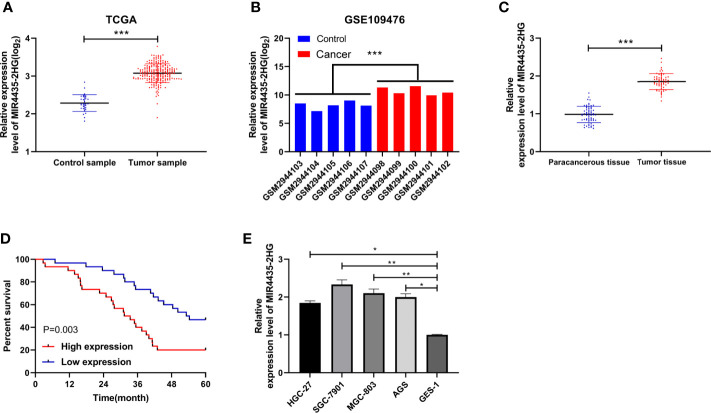
Correlation of MIR4435-2HG in gastric carcinoma with the survival. **(A)** The Cancer Genome Atlas (TCGA) found that MIR4435-2HG elevated in gastric carcinoma. **(B)** MIR4435-2HG in GSE109476 chip of Gene Expression Omnibus (GEO). **(C)** qRT-PCR was applied to test MIR4435-2HG in tumor tissues of patients with gastric carcinoma. **(D)** Kaplan–Meier (K-M) survival analysis of 5-year survival of patients with high and low MIR4435-2HG. **(E)** qRT-PCR detection of MIR4435-2HG in gastric carcinoma cell lines. * indicates p < 0.05; ** indicates p < 0.01; *** indicates p < 0.001.

TCGA and GEO indicated that the expression of MIR4435-2HG was elevated in gastric carcinoma (**Figures 1A, B**). The expression of MIR4435-2HG in clinical gastric carcinoma tissues and cell lines was tested using qRT-PCR, and we found that MIR4435-2HG expression was enhanced in gastric carcinoma (**Figures 1C, E**). Furthermore, the patients were grouped into high and low MIR4435-2HG. Analysis of the correlation of MIR4435-2HG with pathological data revealed that patients with high MIR4435-2HG had lymphatic metastasis, and the probability of TNM III+IV enhanced evidently (**Table 2**). In addition, we followed up the patients for 5 years and found that the high expression of MIR4435-2HG is associated with the poor survival rate of patients (**Figure 1D**). These results suggest that MIR4435-2HG may be a latent marker of gastric carcinoma.

**Table 2:** The relationship between MIR4435-2HG and clinical data of gastric cancer patients.

Factor		MIR4435-2HG relative expression		p-Value
		High expression (n = 30)	Low expression (n = 30)	
Sex				0.284
	Male (n = 38)	17	21
	Female (n = 22)	13	9
Age				0.273
	≥60 years (n = 20)	12	8
	<60 years (n = 40)	18	22
Length of tumor				0.573
	≥5 cm (n = 18)	10	8
	<5 cm (n = 32)	20	22
Lymph node metastasis				0.010
	Positive (n = 12)	10	2
	Negative (n = 48)	20	28
TNM staging				0.017
	I+II staging (n = 23)	16	7
	III+IV staging (n = 37)	14	23

### The Depletion of MIR4435-2HG Could Repress Proliferation, Migration, Invasion, and EMT in Gastric Carcinoma Cells

**Figure 2:**
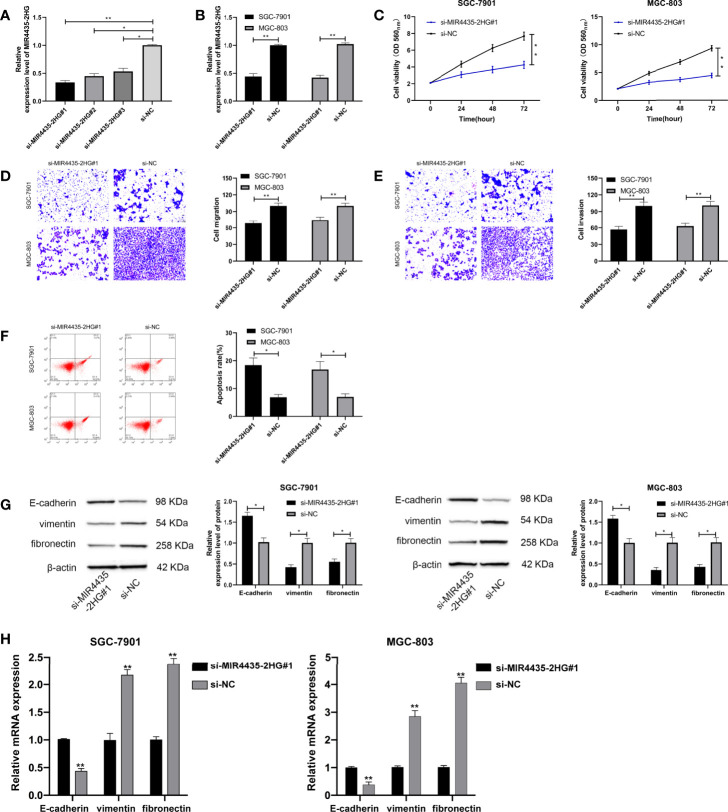
Role of MIR4435-2HG inhibition on growth, metastasis, and epithelial–mesenchymal transition (EMT) of gastric carcinoma cells. **(A)** qRT-PCR was applied to test MIR4435-2HG after constructing si-MIR4435-2HG in SGC-7901 and MGC-803 cells. **(B)** qRT-PCR was applied to test MIR4435-2HG in cells transfected with si-MIR4435-2HG#1 in SGC-7901 and MGC-803 cells. **(C)** Cell Counting Kit-8 (CCK-8) test was applied to test the proliferation of gastric carcinoma cells transfected with si-MIR4435-2HG#1 in SGC-7901 and MGC-803 cells. **(D, E)** Transwell test was applied to test the invasion and migration of gastric carcinoma cells transfected with si-MIR4435-2HG#1 in SGC-7901 and MGC-803 cells. **(F)** Flow cytometry was applied to test the apoptosis of gastric carcinoma cells transfected with si-MIR4435-2HG#1 in SGC-7901 and MGC-803 cells. **(G)** Western blotting experiment was applied to test the changes of EMT protein in cells. **(H)** qRT-PCR was applied to test the mRNA changes of EMT markers in cells in SGC-7901 and MGC-803 cells. * indicates p < 0.05; ** indicates p < 0.01.

Furthermore, MIR4435-2HG interfering RNA (**Figure 2A**) was constructed, and si-MIR4435-2HG#1 with significant effectiveness was selected to transfect into SGC-7901 and MGC-803 cells (**Figure 2B**). MTT assay demonstrated that the proliferation of cells transfected with si-MIR4435-2HG#1 was evidently hindered compared with that transfected with si-NC (**Figure 2C**). Transwell assay revealed that the invasion (**Figure 2D**) and migration (**Figure 2E**) of SGC-7901 and MGC-803 cells transfected with si-MIR4435-2HG#1 were evidently hindered compared with those transfected with si-NC. Flow cytometry revealed that si-MIR4435-2HG#1 induced apoptosis of SGC-7901 and MGC-803 cells (**Figure 2F**). In addition, Western blotting (WB) and qRT-PCR analysis showed that E-cadherin protein was elevated, and that vimentin and fibronectin proteins were hindered after transfection of si-MIR4435-2HG#1 (**Figures 2G, H**), suggesting that decline of MIR4435-2HG could hinder the progression of EMT.

### MIR4435-2HG Acted as a Sponge of MiR-138-5p

**Figure 3:**
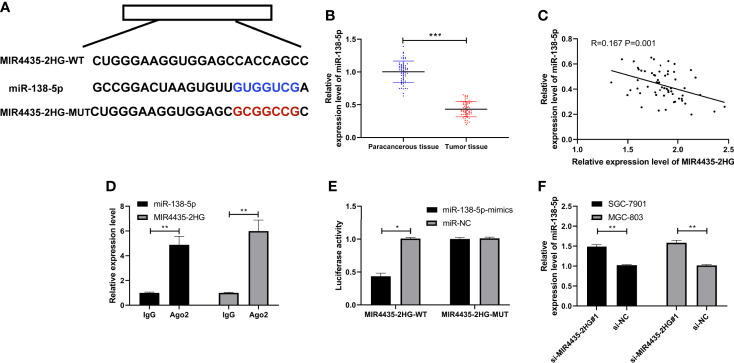
MIR4435-2HG bind to miR-138-5p. **(A)** Specific binding sites of MIR4435-2HG with miR-138-5p. **(B)** qRT-PCR detection of miR-138-5p in gastric carcinoma patients. **(C)** Pearson’s test analyzed the correlation of MIR4435-2HG with miR-138-5p in gastric carcinoma patients. **(D)** RNA immunoprecipitation (RIP) experiment confirmed that MIR4435-2HG combined with miR-138-5p in SGC-7901 and MGC-803 cells. **(E)** Double luciferase report confirmed that MIR4435-2HG combined with miR-138-5p in SGC-7901 and MGC-803 cells. **(F)** MiR-138-5p in gastric carcinoma cells transfected with si-MIR4435-2HG#1 was tested using qRT-PCR in SGC-7901 and MGC-803 cells. * indicates p < 0.05; ** indicates p < 0.01; *** indicates p < 0.001.

LncRNA can interact with miRNA to change tumor growth. To further confirm the mechanism underlying MIR4435-2HG-mediated gastric carcinoma, we predicted the miRNA that can specifically bind to MIR4435-2HG. LncBase (24) and starBase (25) revealed that there are potential binding sites of miR-138-5p with MIR4435-2HG (**Figure 3A**). MiR-138-5p declined in gastric carcinoma (**Figure 3B**), and miR-138-5p expression is negatively correlated with MIR4435-2HG expression (**Figure 3C**). In addition, MIR4435-2HG and miR-138-5p were precipitated by Ago2 (**Figure 3D**), and the luciferase activity of MIR4435-2HG-WT was inhibited by miR-138-5p-mimics (**Figure 3E**). qRT-PCR indicated that miR-138-5p was elevated after transfection with si-MIR4435-2HG#1 in SGC-7901 and MGC-803 cells (**Figure 3F**), suggesting that MIR4435-2HG can target miR-138-5p.

### MiR-138-5p Targeted Sox4

**Figure 4:**
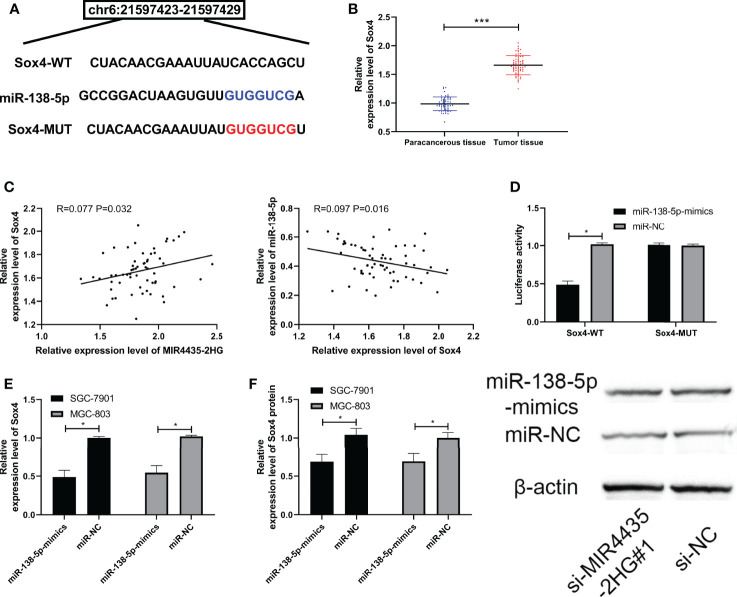
miR-138-5p targeted Sox4. **(A)** Specific binding sites of miR-138-5p and Sox4. **(B)** qRT-PCR was applied to test Sox4 in gastric carcinoma patients. **(C)** Pearson’s test was applied to test the correlation of Sox4 with MIR4435-2HG and miR-138-5p. **(D)** Double luciferase report confirmed that MIR4435-2HG combined with miR-138-5p in SGC-7901 and MGC-803 cells. **(E, F)** qRT-PCR and Western blotting (WB) were applied to test miR-138-5p in gastric carcinoma cells transfected with si-MIR4435-2HG#1 in SGC-7901 and MGC-803 cells. * indicates p < 0.05; *** indicates p < 0.001.

Many reports have confirmed that miRNA hinders the growth of tumor cells by targeting downstream genes. We predicted the downstream target of miR-138-5p through TargetScan (26), miRDB (27), starBase, and miRTarBase (28) and found that there is a potential binding site of Sox4 with miR-138-5p (**Figure 4A**). The expression of Sox4 was upregulated in gastric carcinoma patients (**Figure 4B**), negatively correlated with miR-138-5p, and positively correlated with MIR4435-2HG (**Figure 4C**). The luciferase activity of Sox4-WT was suppressed by miR-138-5p-mimics (**Figure 4D**), and qRT-PCR and WB detection revealed that Sox4 protein and mRNA in gastric carcinoma transfected with miR-138-5p-mimics were obviously inhibited in SGC-7901 and MGC-803 cells (**Figures 4E, F**), indicating that miR-138-5p can target Sox4.

### MIR4435-2HG Inhibited EMT of Gastric Carcinoma by Controlling MiR-138-5p/Sox4 Axis

**Figure 5:**
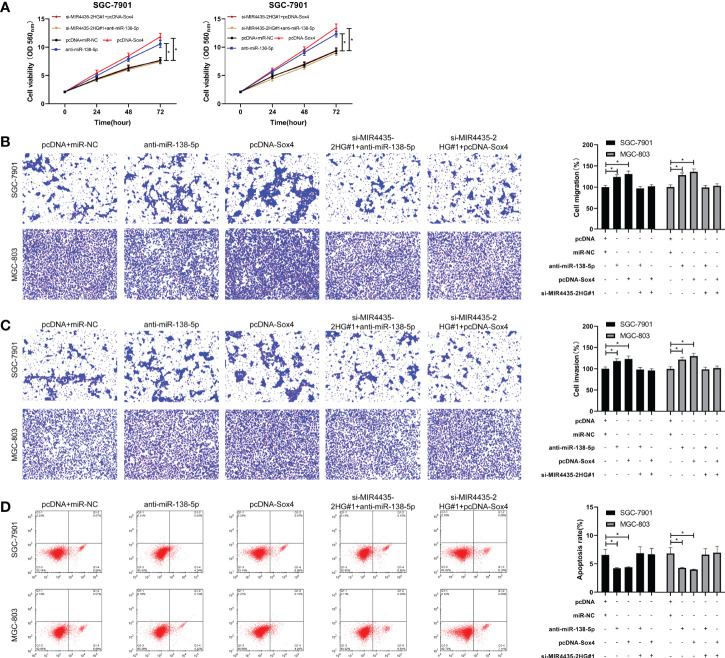
MIR4435-2HG inhibited the growth, metastasis, and epithelial–mesenchymal transition (EMT) of gastric carcinoma cells by mediating miR-138-5p/Sox4 axis. **(A)** Cell Counting Kit-8 (CCK-8)-8 test was applied to test cell proliferation after co-transfection of si-MIR4435-2HG#1 with anti-miR-138-5p and pcDNA-Sox4 in SGC-7901 and MGC-803 cells. **(B, C)** Transwell test was applied to test cell invasion and migration after co-transfection of si-MIR4435-2HG#1 with anti-miR-138-5p and pcDNA-Sox4 in SGC-7901 and MGC-803 cells. **(D)** Flow cytometry was applied to test the cell apoptosis after co-transfection of si-MIR4435-2HG#1 with anti-miR-138-5p and pcDNA-Sox4 in SGC-7901 and MGC-803 cells. Western blotting (WB) was applied to test E-cadherin, vimentin, and fibronectin after co-transfection of si-MIR4435-2HG#1 with anti-miR-138-5p and pcDNA-Sox4 in SGC-7901 and MGC-803 cells. * indicates p < 0.05.

**Figure 6:**
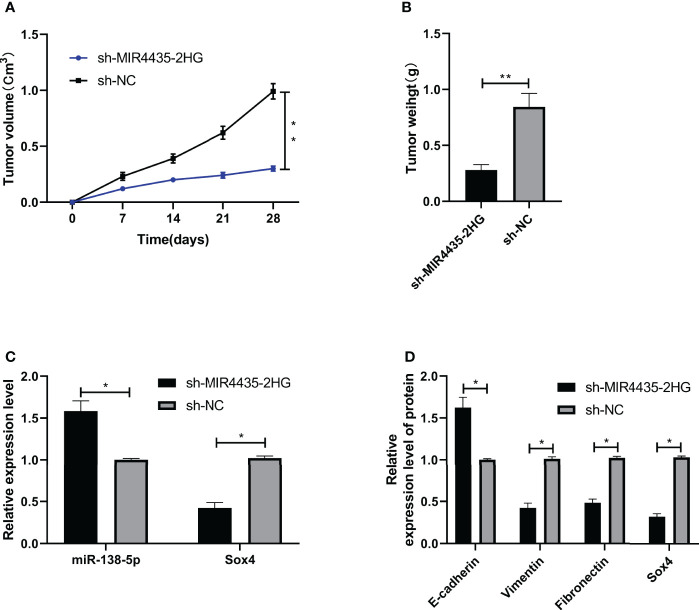
MIR4435-2HG inhibited epithelial–mesenchymal transition (EMT) of gastric carcinoma through miR-138-5p/Sox4 axis. **(A, B)** sh-MIR4435-2HG inhibited the growth of gastric carcinoma in nude mice tumors. **(C)** qRT-PCR was applied to test miR-138-5p and Sox4 in nude mice tumors. **(D)** Western blotting (WB) was applied to test E-cadherin, vimentin, fibronectin, and Sox4 protein in nude mice tumor. * indicates p < 0.05; ** indicates p < 0.01.

si-MIR4435-2HG#1 was co-transfected with anti-miR-138-5p and pcDNA-Sox4 to determine that MIR4435-2HG controls EMT in gastric carcinoma cells through miR-138-5p/Sox4 axis. The result showed that the proliferation (**Figure 5A**), invasion (**Figure 5B**), and migration (**Figure 5C**) of SGC-7901 and MGC-803 cells were hindered after transfection of anti-miR-138-5p and pcDNA-Sox4 (**Figure 5D**). In addition, nude mice experiments revealed that the tumor volume and mass of nude mice declined after injection of sh-MIR4435-2HG (**Figures 6A, B**), and qRT-PCR indicated that miR-138-5p elevated and Sox4 declined after injection of sh-MIR4435-2HG (**Figure 6C**). Furthermore, WB detection also confirmed that E-cadherin protein elevated while vimentin, fibronectin, and Sox4 protein declined (**Figure 6D**), suggesting that MIR4435-2HG can inhibit EMT in gastric carcinoma cells by controlling miR-138-5p/Sox4 axis.

## Discussion

As a common malignancy in the clinic, the incidence and mortality of gastric carcinoma are continuously increasing (29). EMT refers to the biological development of epithelial cells from mesenchymal phenotype cells *via* specific transition (30). The previous investigations have suggested that EMT plays crucial roles in tissue reconstruction, embryonic development, chronic inflammation, and tumor metastasis in various biological processes (31). In this research, we identified that MIR4435-2HG was elevated in gastric carcinoma, and the decline of MIR4435-2HG could hinder EMT of gastric carcinoma cells, which contributed to the progression of gastric carcinoma.

MIR4435-2HG is located on human chromosome 2q13, which is also called LINC00978. The initial exploration showed that MIR4435-2HG was enhanced in breast carcinoma with poor prognosis (32), and others found that MIR4435-2HG declined in osteoarthritis and regulated the proliferation and apoptosis of chondrocytes (33). It has been reported that MIR4435-2HG is involved in the modulation of growth gastric cancer by the activation of Wnt/β-catenin signaling (21). Database analysis indicated that MIR4435-2HG was enhanced in gastric carcinoma, and the survival of patients with high MIR4435-2HG declined. MIR4435-2HG may be a latent prognostic marker of gastric carcinoma. Our data provide evidence of the clinical significance of MIR4435-2HG in gastric carcinoma, and the diagnostic and prognostic values should be evaluated by more clinical studies. In order to further assess the function of MIR4435-2HG in gastric carcinoma, we performed *in vitro* experiments. The results revealed that the cell growth, metastasis, and EMT were hindered after MIR4435-2HG depletion. These data provide new evidence of the critical function of MIR4435-2HG in the regulation of gastric carcinoma progression, especially indicating the innovative role of MIR4435-2HG in the modulation of EMT of gastric carcinoma.

LncRNA acted as a sponge of miRNAs to control miRNAs and their downstream targeted genes and to inhibit EMT in tumor cells (34, 35). For instance, Liang et al. (36) indicated that DLX6-AS1/miR-204-5p/OCT1 positive feedback loop accelerated gastric carcinoma progression and EMT; and other studies (37) showed that lncRNA SNHG7 participated in the proliferation, migration, invasion, and EMT of non-small cell lung carcinoma by controlling miR-449a/TGI F2 axis. Therefore, we predicted the latent miRNAs of MIR4435-2HG, and we found that miR-138-5p had specific binding sites with MIR4435-2HG through LncBase and starBase. MiR-138-5p is differentially expressed in various tumors, such as lung carcinoma (38), colon carcinoma (39), cervical carcinoma (40), and pancreatic carcinoma (41). And some researchers have found that (42) miR-138-5p can hinder the proliferation and invasion of gastric carcinoma by mediating epidermal growth factor receptor (EGFR). Our data suggested that the expression of miR-138-5p was reduced in gastric carcinoma and that the expression of miR-138-5p was negatively correlated with that of miR-4435-2HG, suggesting that there may be a targeted correlation with miR-138-5p and miR-4435-2HG. And we also conducted RIP, double luciferase report assay, and qRT-PCR experiment to confirm this conclusion. These results indicate the novel correlation of MIR4435-2HG with miR-138-5p in the regulation of gastric carcinoma progression, presenting the new mechanism of MIR4435-2HG-mediated malignant development of gastric carcinoma.

The classic mechanism of miRNAs is that miRNAs participate in tumorigenesis by regulating downstream target genes. In this study, we identified miR-138-5p as the target gene of MIR4435-2HG. We predicted the downstream target gene of miR-138-5p through TargetScan, miRDB, starBase, and miRTarBase. As a result, Sox4 has potential binding sites with miR-138-5p. Sox4 is involved in the regulation of embryonic development and the determination of cell fate (43, 44). Previous studies have found that (45) Sox4 contributes to EMT induced by TGF-β in gastric carcinoma cells. Double luciferase report experiment was conducted to test the gastric carcinoma cells transfected with miR-138-5p-mimics, and miR-138-5p could target Sox4. To verify the correlation of Sox4 with miR-138-5p and MIR4435-2HG, we analyzed the expression of Sox4 in gastric carcinoma patients and found that Sox4 was elevated. The expression of Sox4 was positively correlated with MIR4435-2HG and negatively correlated with miR-138-5p, suggesting that MIR4435-2HG may regulate EMT in gastric carcinoma cells through miR-138-5p/Sox4 axis. Our data provide the new insight into the mechanism by which MIR4435-2HG contributes to EMT and malignant progression of gastric carcinoma by targeting miR-138-5p/Sox4 axis. The previous studies have shown the critical function of Sox4 in the modulation of EMT, and we found that MIR4435-2HG enhanced Sox4 expression by targeting miR-138-5p. Accordingly, MIR4435-2HG may regulate vimentin, E-cadherin, or fibronectin expression by miR-138-5p/Sox4 axis, and more investigation should be performed to confirm it.

Moreover, we co-transfected si-MIR4435-2HG with anti-miR-138-5p and pcDNA-Sox4 and found that the proliferation, invasion, migration, apoptosis, and EMT of gastric carcinoma transfected with anti-miR-138-5p and pcDNA-Sox4 were reversed after co-transfection with si-MIR4435-2HG. In addition, *in vivo* experiments revealed that the tumor mass and volume in nude mice were evidently inhibited, and that miR-138-5p enhanced and Sox4 declined after injection of sh-MIR4435-2HG. E-cadherin was elevated, and vimentin and fibronectin were inhibited, which further confirmed our research conclusion. However, there are still some limitations in this study. First, we only detected the tumor tissues of patients with gastric carcinoma and did not further detect other samples of patients. The diagnostic value of MIR4435-2HG in gastric carcinoma is unknown. Second, the progression of gastric carcinoma may be affected through other mechanisms, which are not clear and need to be studied. Meanwhile, MIR4435-2HG has been reported to target miR-22-3p, miR-28-3p, miR-128-3p, and miR-1224-3p in the previous investigations, but the correlation of MIR4435-2HG with these miRNAs in the modulation of gastric carcinoma is still elusive and should be explored in future investigations. Therefore, we will add our related experiments to further improve our conclusions.

To sum up, MIR4435-2HG is elevated in gastric carcinoma cells and contributes to the growth, metastasis, and EMT of gastric carcinoma cells by mediating miR-138-5p/Sox4 axis, which may be a latent treatment target in clinic.

## Data Availability Statement

The original contributions presented in the study are included in the article/supplementary material. Further inquiries can be directed to the corresponding author.

## Ethics Statement

The studies involving human participants were reviewed and approved by Cangzhou Central Hospital. The patients/participants provided their written informed consent to participate in this study.

## Author Contributions

L-FG and WL designed the study. Y-GL and CZ performed experiments. W-NG collected and analyzed the data. LW wrote the manuscript. All authors contributed to the article and approved the submitted version.

## Conflict of Interest

The authors declare that the research was conducted in the absence of any commercial or financial relationships that could be construed as a potential conflict of interest.

## Publisher’s Note

All claims expressed in this article are solely those of the authors and do not necessarily represent those of their affiliated organizations, or those of the publisher, the editors and the reviewers. Any product that may be evaluated in this article, or claim that may be made by its manufacturer, is not guaranteed or endorsed by the publisher.
